# Trans-regional migration of the beet armyworm, *Spodoptera exigua* (Lepidoptera: Noctuidae), in North-East Asia

**DOI:** 10.1371/journal.pone.0183582

**Published:** 2017-08-25

**Authors:** Xiaowei Fu, Hongqiang Feng, Zhongfang Liu, Kongming Wu

**Affiliations:** 1 State Key Laboratory for Biology of Plant Diseases and Insect Pests, Institute of Plant Protection, Chinese Academy of Agricultural Sciences, Beijing, P.R., China; 2 Institute of Plant Protection, Henan Academy of Agricultural Sciences, Zhengzhou, P.R., China; 3 Institute of Plant Protection, Shanxi Academy of Agricultural Sciences, Taiyuan, P.R., China; Pennsylvania State University, UNITED STATES

## Abstract

The beet armyworm, *Spodoptera exigua* (Hübner) (Lepidoptera: Noctuidae), is a serious polyphagous insect pest worldwide. This species is known as a long-distance migrant, and previous studies on its migration have been mostly carried out in regions where it can overwinter. However, what pattern of seasonal migration this species exhibits in regions where it cannot overwinter (i.e., the ‘summer breeding region’) remains unknown. Here, we present data from 14-years of monitoring on a small remote island located in the center of the Bohai Strait, in northern China, by means of searchlight trapping and ovarian dissection. We found that the population size of this overseas migration varied significantly among years, with very large migrations in 2005, 2007, 2009, and 2014 that resulted in annual total catches of more than ten thousand individuals. In addition, nightly catches exhibited a significant inter-month variability, with the vast majority of *S*. *exigua* moths being trapped in August and September, (81.1 ± 3.6%), making *S*. *exigua* one of the most frequently encountered species in that period. The mean time from the earliest trap capture to the latest capture within a given year was 113 ± 22 d (range 57 d [2003] to 138 d [2008]). The sex ratio (females: males) was significantly less than 1:1 in each month, but the proportion of females showed an upward trend from June to October. The majority of trapped females in summer were mated (94.4 ± 10.7% in June, 80.0 ± 6.4% in July) and sexually mature (88.9 ± 11.1% in June, 61.8 ± 12.3% in July), suggesting the onset of mating and/or sexual maturation does not terminate the migration behavior in this species. These findings provide a good starting point for study of the trans-regional migration of *S*. *exigua* across different climate zones.

## Introduction

Since the late 1980s the beet armyworm, *Spodoptera exigua* (Hübner) (Lepidoptera: Noctuidae), has been a destructive pest attacking >90 plant species in Asia, including several major crops, such as sugar beet, cotton, soybean, and potatoes [1–3]. *S*. *exigua* is one of the three most important pest species in the genus *Spodoptera*, and the young larvae always feed on terminal clusters, seedlings, and stems of host crops [4–5]. Successful management of *S*. *exigua* is complicated by its broad host range, rapid growth rate, high fecundity, strong capacity for long-duration flight, and the rapid evolution of resistance to pesticides [6–11]. Serious outbreaks of this pest in many regions of Asia, Africa, Europe, and America have been reported in the past two decades [7, 12–13].

**Fig 1:**
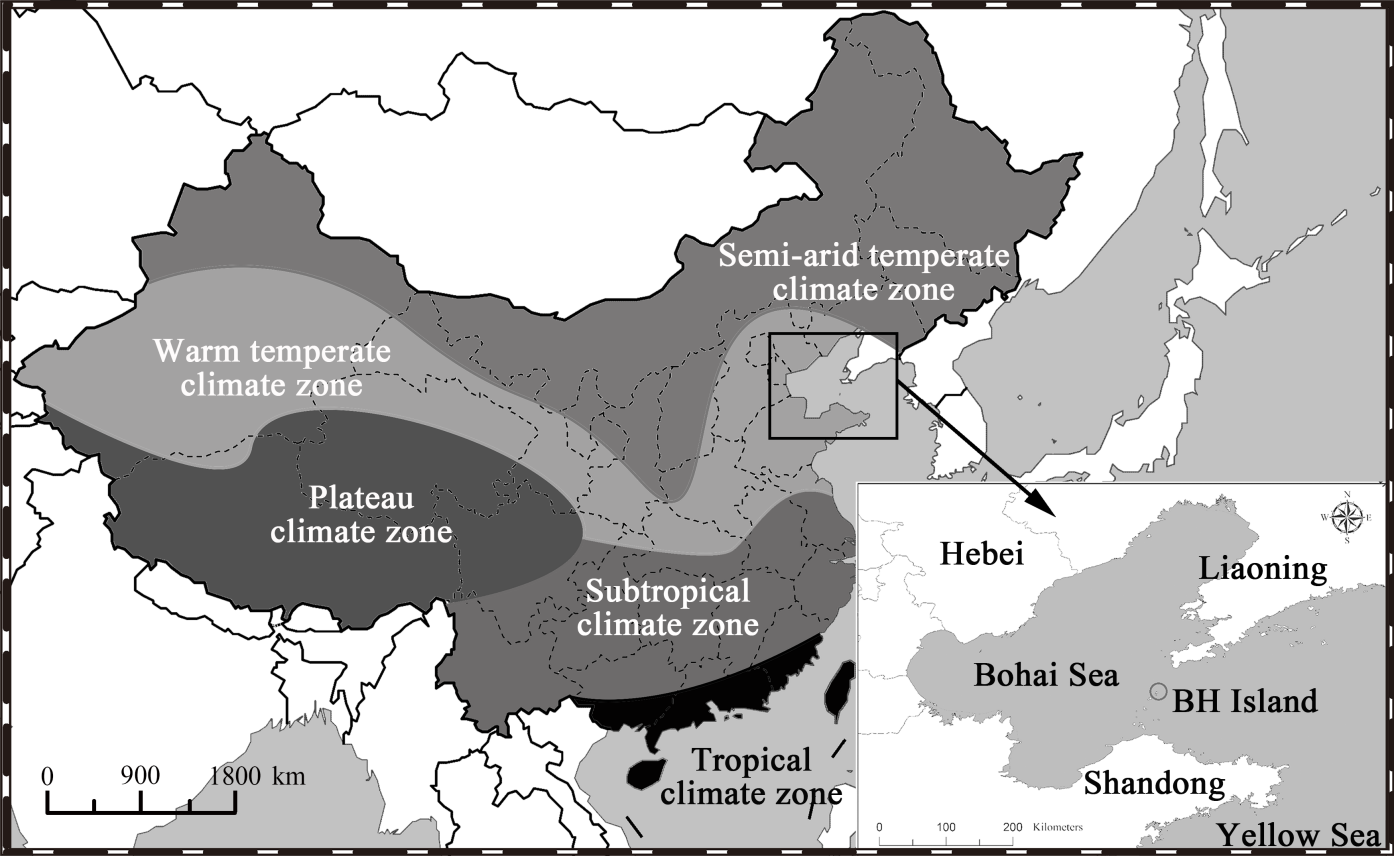
Maps showing different climate zones in China (left) and the position of BeiHhuang Island, the searchlight trapping site (right), relative to the Bohai and yellow seas.

Insects have evolved two general strategies to cope with habitat changes: diapause and migration, which have been referred to as the ‘here later’ and ‘there now’ strategies [14]. Long-distance migration across different climate zones plays a key role in the life-history of many insects and is an important reason for the sudden outbreaks of some crop pests [15]. In 1960s, *S*. *exigua* was first reported as a migrant in British Isles [16], and since then the synoptic chart analyses, light-trap monitoring, and radar observations have shown that *S*. *exigua* undertakes long-distance migration in Asia, Europe, and North America [16–20]. In east Asia, 4 to 11 generations of *S*. *exigua* occur annually, increasing as the growing degree-days (GDD) increase from north to south [5, 7]. The occurrence range of *S*. *exigua* in east Asia can be divided into three zones: (1) the ‘year-round breeding region’ (< 25°N), mainly including the tropical climate zone (TCZ) and the southern part of subtropical climate zone (STCZ). In this region, *S*. *exigua* can breed year-round without diapause [9]. (2) the ‘overwintering region’ (25–35°N), mainly ranging from the northern part of the STCZ to the middle part of warm temperate climate zone (WTCZ). In this region, *S*. *exigua* overwinter as pupae in the soil [9]. (3) the ‘summer breeding region,’ mainly including regions north of 38°N, and *S*. *exigua* cannot overwinter in this region [7, 9, 21–22] (Fig 1).

Previous studies on the migration of *S*. *exigua* in east Asia have mostly been carried out in regions where it can overwinter (i.e., the ‘year-round breeding region’ and the ‘overwintering region’) [7, 9]. However, what pattern of seasonal migration this species exhibits in regions where it cannot overwinter (i.e., the ‘summer breeding region’) remains unknown. Considering the likely poleward expansion of many insects expected under current global warming scenarios [23–27], it is critical to enhance our knowledge of the trans-regional migration of *S*. *exigua*. In the current study, searchlight trapping and ovarian dissection were combined to monitor the wind-borne migration of *S*. *exigua* over 14 consecutive years on Beihuang (BH, 38°24′N; 120°55′E) Island, which is located in the center of the Bohai Strait in northern China. Findings from this study will be helpful for understanding the occurrence regularity of *S*. *exigua* migration across different climate zones.

## Materials and methods

### Ethics statement

No specific permits were required for the described field studies, since the study was conducted in an experiment station (Shandong province) owned and maintained by the Institute of Plant Protection, Chinese Academy of Agricultural Sciences, and all investigators of this study are employed by this institute. These field studies did not involve endangered or protected species.

### Study area

The current study was carried out at BH (Fig 1), a wild experimental station of Chinese Academy of Agricultural Sciences. BH is the northernmost island of Shandong Province and is in the center of the Bohai Strait. BH (2.5 km^2^) is ≈ 40 km and ≈ 60 km from the mainland to the north and to the south, respectively.

### Searchlight trapping

**Fig 2:**
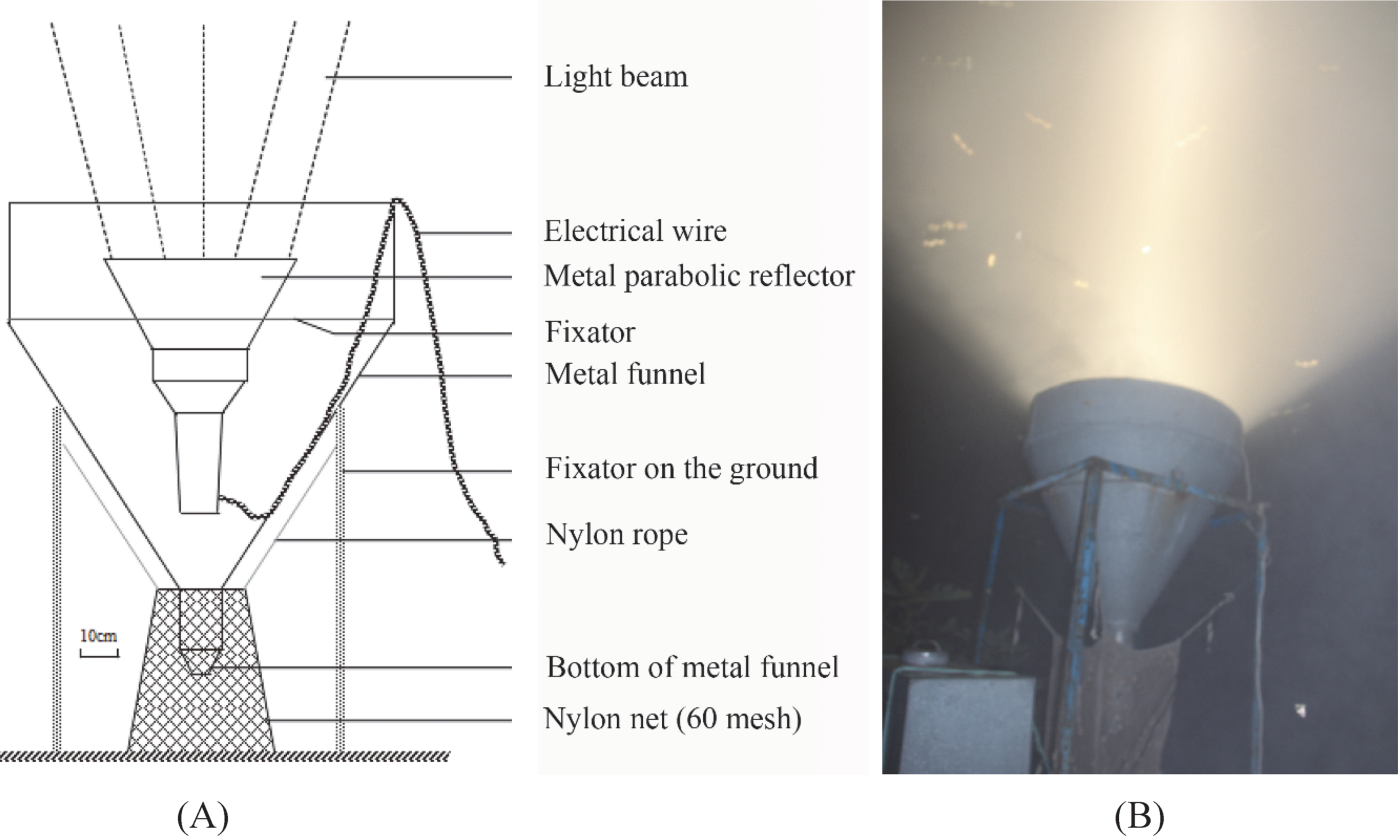
Schematic diagram (A) and nocturnal view (B) of the searchlight trap.

A vertically oriented searchlight (model DK.Z.-J1000B/t, 65.2 cm in diameter, 70.6 cm in height and approximately 30° in spread angle; Shanghai Yaming Lighting Co.Ltd., Shanghai, China. Fig 2) was used as a light trap to attract and collect migrating insects from high altitudes (up to ≈ 500 m above ground level) [19]. The trap was equipped with a 1,000-W metal halide lamp (model JLZ-1000BT; Shanghai Yaming Lighting Co. Ltd., Shanghai, China), which produced a vertical beam of light with a luminous flux of 105,000 lm, a color temperature of 4,000 K, and a color rendering index of 65.

The searchlight trap was turned on at sunset and off at sunrise on all nights from April to October 2003–2016. Incomplete data sets resulting from power cuts or heavy rains were excluded from the analysis. Trapped insects were collected with a nylon net bag (60 mesh) beneath the trap, which was changed manually every 2 h each night. Trapped insects were held at -20°C for 4 h before being identified and the females of *S*. *exigua* dissected.

There are some pine trees and graminaceous weeds on BH, but no arable land or crops that are *S*. *exigua* hosts. To investigate whether any *S*. *exigua* moths were produced on BH itself, visual observations [28] were carried out daily to detect larvae of this species on any potential wild host plants from spring through to autumn 2003–2016.

### Ovarian dissection

From 2005 to 2009, a subsample of 20 *S*. *exigua* females (or all available females if the nightly total capture of female *S*. *exigua* was < 20) was randomly taken from moths trapped each night and dissected under a stereomicroscope (model JNOEC-Jsz4; Motic China Group Co.Ltd., Xiamen, China) to determine the mating rate, mating occurrences and ovarian development level.

The ovarian development level of female *S*. *exigua* moths was estimated as described by Wang et al. [29]: Level 1, undeveloped thin and short oviducts, and semitransparent milky white ovarioles; Level 2, developing yellow eggs appeared in elongated ovarioles but did not reach oviducts; Level 3, mature eggs fully filled in not only the well-developed ovarioles but also lateral and median oviducts; Level 4: a proportion of eggs laid and interspaces appeared between mature eggs. Level 5: the shortest shrunk oviducts and ovarioles with almost no eggs. Female moths with ovarian development levels 1–2 were regarded as “sexually immature individuals,” while moths with ovarian development levels 3–5 were regarded as “sexually mature individuals” [29]. These ovarian developmental index values were averaged to generate monthly means of ovarian development. In addition, mating rates (mating per female) and mating occurrence level (% non-virgin females) were determined by the number of spermatophores in the spermatheca of female moths.

### Meteorological and map data

Daily maximum and minimum surface air temperature on BH were obtained from the National Meteorological Information Center of China (http://www.nmic.cn/). The geographical map of China with administrative division at province level (1: 9,000,000) was downloaded from the National Geomatics Center of China (NGCC; http://ngcc.sbsm.gov.cn/). Times in this paper are all given as Beijing time (GMT + 0800 h).

### Data analysis

Dates used for trap catches indicate the period from sunset of that day to sunrise of the next day, and means of moth captures are all given as M ± SEM. Differences in the number of *S*. *exigua* captured in the searchlight trap, the monthly mean proportion of females, mated females, and sexually mature females were analyzed using generalized linear mixed models (GLMM), with month as the fixed effect and year as the random effect [30]. All proportion data were first arcsine square root transformed before analysis. If ANOVA indicated a significant difference among months, Tukey’s HSD (honestly significant difference) test was used to distinguish significantly different monthly means. Chi-square tests were used to test whether sex ratios differing from 1: 1 (females: males) for months from 2005 to 2009. Differences between the mean proportion of mated and unmated females and between sexually mature and immature females were compared by using *t*-tests.

In order to distinguish strong or weak invasion years, the annual total catches of *S*. *exigua* were analyzed by hierarchical cluster analysis, with the clustering distance of ‘Euclidean Distance’ and the clustering method of ‘Nearest-neighbor Method’ [31].

To delineate trends of sex ratio, mating rate, and ovarian development levels between June and October, the proportions of females, mated females, and sexually mature females from June to October were analyzed versus time by logistic regression:

$$y=\frac{K}{1+ae^{bx}}$$

where *K* is the parameter of saturation, *a* is model coefficient, *b* is the relative growth rate, and *x* is different months.

To delineate the occurrence status of trapped *S*. *exigua* in each month, the index of occurrence (*O*) was introduced and calculated:

$$O=\frac{p}{n}\times 100\%$$

where *p* is the number of nights in which *S*. *exigua* moths were trapped in a month, and *n* is the number of nights in which all insect species were trapped in a month [32]. Occurrence status of *S*. *exigua* captured in the searchlight trap in each month was determined by the following criteria: as a coincidental species with *O* = 0% - 25%, as an accessory species with *O* = 25% - 50%, and as a constant species with *O* = 50% - 100% [33].

In order to explain the variation of dates of first capture of *S*. *exigua* in each year, the growing degree-days (GDD) of this species was introduced and calculated [34]:

$$GDD=\frac{daily\;maximum\;temperature-daily\;minimum\;temperature}{2}-13.82{}^{\circ}C$$

where 13.82°C is the base temperature for development of *S*. *exigua* moths as determined by Yan et al. [35] and Zhang [36]. Cumulative GDD were calculated from January 1 by totaling the daily GDD values.

All statistical analysis was performed by the software of Data Processing System (DPS, V9.5) [37–38], except for sex ratio, which was analyzed by SAS software [39].

## Results

### Annual pattern of *S*. *exigua* migration

**Fig 3:**
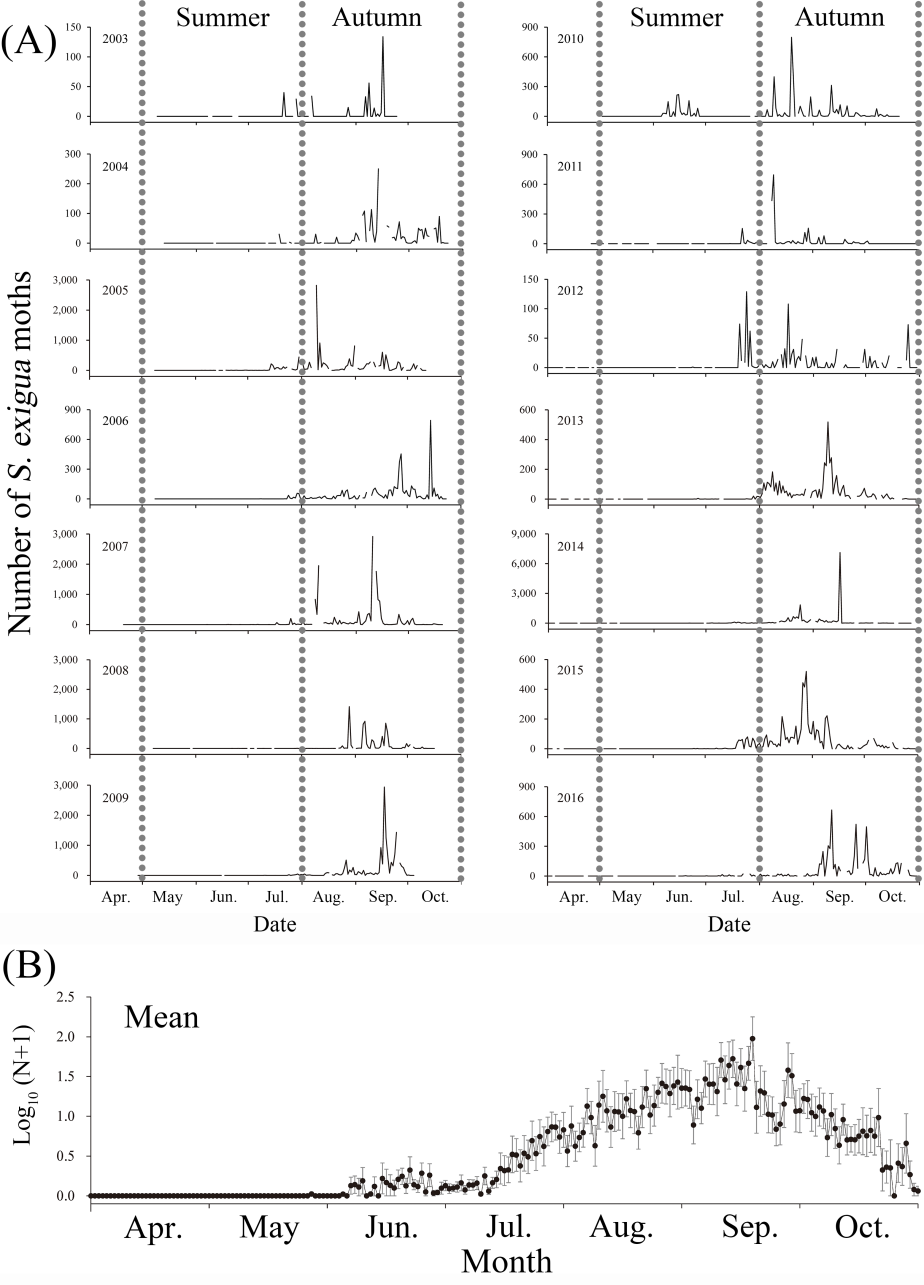
Nightly number (A) and the mean logarithm numbers (B) of *Spdoptera exigua* moths captured in the searchlight trap on BeiHhuang Island from April to October 2003–2016. Note: vertical bars in (B) represent standard errors.

During the 14 years of monitoring, no *S*. *exigua* larvae were found on BH by field investigations, although some wild graminaceous weeds suitable as host plants were present. However, *S*. *exigua* moths were regularly captured in the searchlight trap from May to October every year on BH (Fig 3A; S1 Fig), which strongly suggests that these moths frequently immigrate from the mainland rather than emerging locally, a distance of at least 40–60 km.

From 2003–2016, the strength of this over-sea migration showed significant inter-year variability (*F*_13, 2297_ = 3.37, P < 0.001; S1 Table), with a significant month × year interaction (*F*_72, 2297_ = 2.28, P < 0.001; S1 Table). The results of hierarchical cluster analysis (*R*^2^ = 0.98, Pseudo_F = 199.41, df_1_ = 3, df_2_ = 10, P < 0.001) indicated that the annual pattern of *S*. *exigua* migration across the sea could be divided into four groups (S2 Table): (1) the highest migration that took place in 2014, with a total catch of 18492 individuals; (2) other mass migration years in 2005, 2007 and 2009, with the annual total catches reaching 13319, 14075 and 13206 individuals, respectively; (3) moderate migrations in 2006, 2008, 2010, 2013, 2015 and 2016, with the annual total catches of 4532, 6870, 4444, 4514, 5701 and 5326 individuals, respectively; and (4) weak migrations in 2003, 2004, 2011 and 2012, with the annual total catches of 365, 1653, 2165 and 978 individuals, respectively.

### Seasonal pattern of *S*. *exigua* migration

Variation of daily trap catches showed a seasonal pattern in *S*. *exigua* migration. No *S*. *exigua* moths were trapped between November and late May, and the first capture of *S*. *exigua* in a year generally occurred in June, with the earliest individual trapped on 27 May 2008 (Table 1). Subsequently, moths were trapped intermittently at a low density with nightly mean catches generally being fewer than 10 individuals from May to June (Fig 3B). From July to August, *S*. *exigua* adults were frequently trapped in greater numbers, with nightly mean catches exceeding 50 moths. Trap catches of *S*. *exigua* increased to high levels (generally >100 individuals) in late August with high catch levels continuing through late September and annual peak values frequently occurring in mid-September (Fig 3B). After late September, this species was trapped intermittently, and nightly mean catches decreased to nearly zero by late October (Fig 3B).

**Table 1:** Duration and occurrence status of *Spodoptera exigua* moths captured in the searchlight trap on BeiHuang Island from May to October 2003–2016.

Year	Occurrence status ^*a*^							Date of first capture ^*b*^	Date of final capture ^*b*^	Duration (d)	Date of peak catches (n) ^*b*^
	Apr.	May	Jun.	Jul.	Aug.	Sep.	Oct.				
2003								21 Jul. (40)	16 Sep. (134)	57	16 Sep. (134)
2004								18 Jul. (30)	20 Oct. (2)	94	13 Sep. (250)
2005								04 Jun. (1)	08 Oct. (60)	126	09 Aug. (2821)
2006								08 Jul. (1)	20 Oct. (24)	104	14 Oct. (792)
2007								18 Jun. (1)	17 Oct. (6)	121	10 Sep. (2920)
2008								27 May (1)	12 Oct. (2)	138	28 Aug. (1415)
2009								14 Jun. (1)	01 Oct. (1)	109	17 Sep. (2937)
2010								06 Jun. (30)	13 Oct. (16)	129	19 Aug. (798)
2011								15 Jul. (1)	02 Oct. (27)	79	09 Aug. (695)
2012								23 Jun. (1)	25 Oct. (73)	124	24 Jul. (129)
2013								24 Jun. (1)	25 Oct. (1)	123	09 Sep. (518)
2014								19 Jun. (4)	14 Oct. (2)	117	16 Sep. (7128)
2015								24 Jun. (2)	26 Oct. (4)	124	28 Aug. (520)
2016								18 Jun. (1)	28 Oct. (1)	132	11 Sep. (666)

^*a*^ ▇ occurrence index between 50%–100%, ▇ occurrence index between 25%–50%, ▇ occurrence index between 0%–25%.

^*b*^ The numbers of *S*. *exigua* moths captured are given in parentheses next to name of the months.

During the study period (2003–2016), nightly catches of *S*. *exigua* moths showed a significant inter-month variability (*F*_6, 2297_ = 890153.18, P < 0.001; S1B Fig; S1 Table), with a significant month × year interaction (*F*_72, 2297_ = 2.28, P < 0.001; S1 Table). The vast majority of *S*. *exigua* moths being trapped in August and September, (81.1 ± 3.6%), making *S*. *exigua* one of the most frequently encountered species in that period.

**Fig 4:**
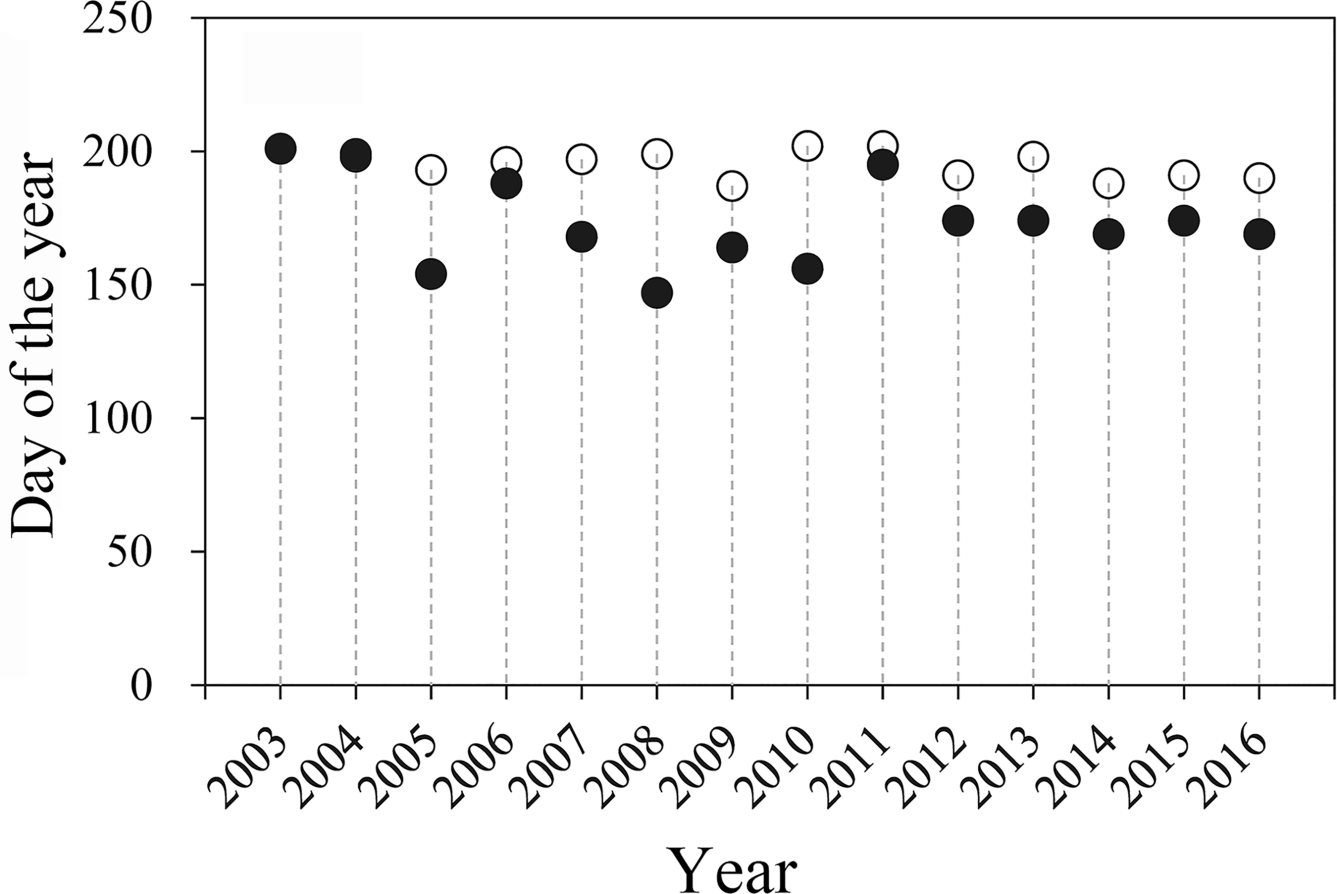
First capture of *Spodoptera exigua* moths in the searchlight trap on BeiHhuang Island from April to October 2003–2016. Note: (○) indicates the predicted date of first capture based on the cumulative growing degree-days (GDD), and (●) indicates the actual date observed by searchlight trap.

During the study period, *S*. *exigua* moths were captured frequently in the searchlight trap and considered a constantly recovered species in August and September (Table 1). In July and October, this species was trapped occasionally and considered a species of secondary abundance (Table 1), while in other months this species occurred only as a coincidental insect (Table 1). During 2003–2016, the mean time from first to last catch of *S*. *exigua* within a year was 113 ± 22 d, with the shortest period being 57 d in 2003, while the longest was 138 d in 2008 (Table 1). Estimated dates of *S*. *exigua* moth emergence based on GDD on BH from 2003 to 2016 (Fig 4) appeared to be late, as actual dates of first capture of this species in the searchlight trap on BH were 22 ± 4 d earlier (Fig 4). This difference provides additional evidence that trapped *S*. *exigua* moths were immigrants from the mainland rather than locally emerged moths.

### Seasonal pattern of sex ratio

**Fig 5:**
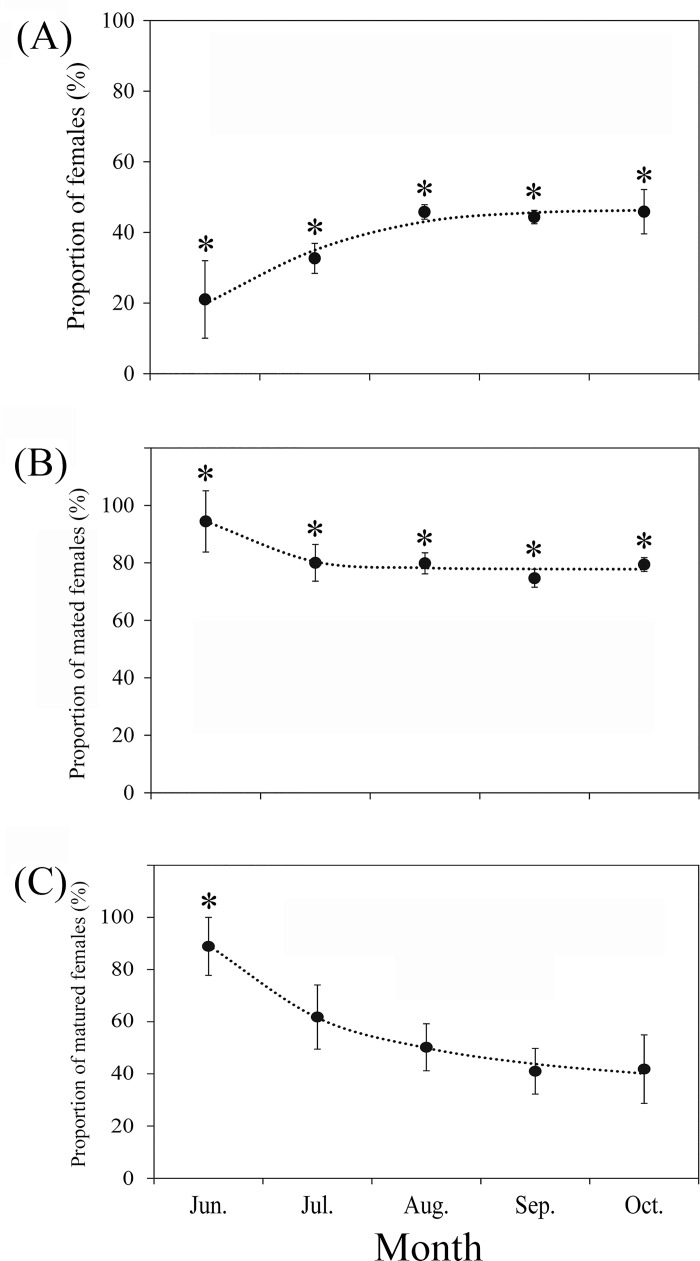
Proportion of females (A), mated females B), and sexually mature females (C) of *Spodoptera exigua* captured in the searchlight trap from June to October 2005–2009. Note: Dots indicate the monthly mean proportion from 2005 to 2009. Vertical bars represent standard errors between years in each month. A single asterisk (*) above a bar in (A) indicates the sex ratio (females: males) was < 1:1 in that month at the 5% level of significance as determined by chi-squared test. A single asterisk (*) above a bar in (B) and (C) indicates there was significant difference between the monthly mean proportion of mated and unmated females, and that of sexually mature and immature females at the 5% level of significance as determined by *t*-test.

From June to October for 2005–2009, most trapped *S*. *exigua* moths were males (June, 79.0 ± 11.0%; July, 67.4 ± 4.2%; August, 54.2 ± 2.1%; September, 55.7 ± 1.9%; October, 54.1 ± 6.3%), and this proportion was significantly higher than that of females in each month (Chi-square tests: June, χ^2^ = 13.07, *df* = 1, P < 0.001; July, χ^2^ = 65.56, *df* = 1, P < 0.001; August, χ^2^ = 202.79, *df* = 1, P < 0.001; September, χ^2^ = 433.21, *df* = 1, P < 0.001; October, χ^2^ = 85.17, *df* = 1, P < 0.001; Fig 5A; S2A Fig).

The mean proportion of females showed significant inter-year variability (*F*_4, 363_ = 4.95, P <0.001; S3 Table) and a significant month × year interaction (*F*_15, 363_ = 1.74, P = 0.043; S3 Table). Although there was no significant inter-month difference (*F*_4, 363_ = 0.67, P = 0.626; S3 Table) in the mean proportion of females from June to October 2005–2009, this ratio showed an upward trend from June to October (logistic regression model: *y* = 0.47 / (1 + 3552.90 * *e*^-1.32x^, *R*^2^ = 0.98, *n* = 5, *F* = 255, P <0.001; Fig 5A).

### Seasonal pattern of mating rate and mating occurrences

From June to October for 2005–2009, the vast majority of trapped *S*. *exigua* females were mated (June, 94.4 ± 10.7%; July, 80.0 ± 6.4%; August, 79.9 ± 3.7%; September, 74.7 ± 3.1%; October, 79.4 ± 2.4%), at numbers significantly higher than that of unmated individuals in each month (*t*-tests: June, *t* = 6.51, *df* = 4, P = 0.003; July, *t* = 4.61, *df* = 8, P = 0.002; August, *t* = 9.38, *df* = 8, P < 0.001; September, *t* = 10.15, *df* = 8, P < 0.001; October, *t* = 15.39, *df* = 8, P < 0.001; Fig 5B; S2B Fig).

The mean proportion of mated females showed a significant inter-year variability (*F*_4, 296_ = 4.24, P = 0.002; S4 Table). Although there was no significant inter-month difference (*F*_4, 296_ = 1.27, P = 0.330; S4 Table) in the mean proportion of mated females from June to October 2005–2009, there was a downward trend in the proportion of mated females from June to October (logistic regression model: *y* = 0.78 / (1–3838.29 * *e*^-1.67x^, *R*^2^ = 0.93, *n* = 5, *F* = 1119, P < 0.001;Fig 5B). In addition, the vast majority of mated females had mated once (87.3 ± 3.1%), more than one-tenth (11.1 ± 2.4%) had mated twice, while only a small proportion had mated three (1.4 ± 0.8%) or four (0.2 ± 0.1%) times, and there were no individuals that had mated more than four times (S4 Fig).

### Seasonal pattern of ovarian development

In June, the vast majority of trapped *S*. *exigua* females were sexually mature (88.9 ± 11.1%), and these numbers were significantly higher than that of sexually immature individuals in the same month (*t* = 3.40, *df* = 4, P = 0.016; Fig 5C; S2C Fig). However, there was no significant difference between the proportion of sexually mature versus immature females in other months (July, 61.8 ± 12.3%, *t* = 1.48, *df* = 8, P = 0.178; August, 50.2 ± 9.0%, *t* = 0.13, *df* = 8, P = 0.904; September, 41.0 ± 8.8%, *t* = 1.44, *df* = 8, P = 0.188; October, 41.8 ± 13.1%, *t* = 1.12, *df* = 8, P = 0.296;) (Fig 5C; S2C Fig; S4 Fig).

The mean proportion of sexually mature females showed a significant inter-year variability (*F*_4, 296_ = 19.09, P <0.001; S5 Table), with a significant month × year interaction (*F*_14, 296_ = 2.77, P <0.001; S5 Table) in the analysis. Although there was no significant inter-month difference (*F*_4, 296_ = 2.80, P = 0.067; S5 Table) in the mean proportion of sexually mature females from June to October 2005–2009, we observed a downward trend from June to October (logistic regression model: *y* = 0.33 / (1–3.91 * *e*^-0.30x^, *R*^2^ = 0.99, *n* = 5, *F* = 589, P < 0.001; Fig 5C).

## Discussion

Daily observations showed that no local *S*. *exigua* larvae were found on BH, which is 40–60 km from the mainland, confirming that the large number of *S*. *exigua* moths trapped there must have been long-distance migrants [40–42]. Our results showed a continuous capture of *S*. *exigua* moths from May to October 2003–2016, and the annual and seasonal pattern of this species migration across the Bohai sea was similar to the previous studies for other Lepidoptera [28, 43–47], Odonata [48], Coleoptera [49], and Hemiptera [50] insects observed in the same location.

The strength of this oversea migration varied significantly between years, and the annual pattern of *S*. *exigua* migration across the Bohai Strait could be divided into four groups: the maximum migration year (2014), the mass migration years (2005, 2007, 2009), the moderate migration years (2006, 2008, 2010, 2013, 2015, 2016), and the weak migration years (2004, 2011, 2012). Although there was no regular monitoring of *S*. *exigua* on the mainland, we could not exclude the possibility that the population fluctuations observed in this study may reflect the occurrence of this pest in the mainland. If so, our monitoring on BH island would have provided good surveillance of this pest. Certainly, weather conditions play an important role in the windborne migration of insects [51–53], and some weather conditions may promote aggregation [44, 48, 53]. However, it may also be that large catches of *S*. *exigua* moths in the current study were due to concentration effects caused by large-scale weather systems, and may not always be an indication of outbreaks of this pest on the mainland, a possibility which merits further study.

The East-Asian monsoon airflows promote the long-distance migration of many insect species [52]. Previous studies of the migration of *S*. *exigua* show that this species has a tendency to head approximately downwind at an acute angle to the wind direction [19]. In early summer, *S*. *exigua* mainly occurs in the Yangtze River Valley of China, from which some may migrate using the prevailing southwesterly winds into northern China [9]. In the current study, *S*. *exigua* moths were regularly captured on BH from late May through to July. Considering the prevailing southerly winds in this season, the early-summer population of *S*. *exigua* might take windborne migration to exploit temporary habitats in northeastern China, where the widely planted sugar beet and soybean play an important role for the survival of their offspring. Similar wind-related orientation behavior has been observed in the summer movements of *Helicoverpa armigera* (Hübner) [54], *Loxostege sticticalis* L. [55], *Spodoptera litura* (F.) [45], *Heliothis viriplaca* (Hüfnagel) [28], and *Apolygus lucorum* (Meyer-Dür) [50] in the same place.

In autumn, northerly winds driven by temperature gradients prevailed from eastern Siberia to northern China, and updraft airflows generally occurred in the northeastern agricultural region of China. This weather system promoted large numbers of the offspring population, which was produced by the summer parental generation, to emigrate from the northeastern agricultural region of China southward across the sea and BH to the mainland in the south. Characteristics of the southward return migration observed in the present study were similar to the windborne migration of *Empoasca fabae* (Harris) [56], *Nilaparvata lugens* (Stål) [57], *Cnaphalocrocis medinalis* Guenée [58], *Agrotis ipsilon* (Rottemberg) [59], *Vanessa atalanta* (L.) [60], *Mythimna separata* (Walker) [61], *H*. *armigera* [43–44], and *Autographa gamma* (L.) [62], and it has been postulated that this behavior facilitates the return migration of these species to the southern overwintering regions.

Many insects migrate pole-ward from their winter habitats (low-latitudes) each spring, to exploit temporary resources where they can breed in summer (high-latitudes) but cannot survive in winter [62–67]. Some studies have suggested that low-latitude winter habitats are the major breeding grounds for migratory insects, because the pole-ward migration always results in a population sink as their offspring seldom return: a phenomenon known as the ‘Pied Piper’ effect [68–69]. However, this idea makes little evolutionary sense [62, 65, 70]. In this study, our results showed that the trapped autumn population (offspring generation produced by the summer population) was 44.2 ± 17.5 times bigger than the summer population (Table 2), which suggests that *S*. *exigua*, and possibly other migratory insect species, should be reclassified as an obligate migrant whose primary populations inhabit high-latitude temperate zones and are connected with low-latitude winter-breeding sites that represent annual bottlenecks. These results provide an important step toward quantifying the evolutionary drivers of long-range insect migrations.

**Table 2:** Population abundance and seasonal increase of *Spodoptera exigua* measured by searchlight trap on BeiHuang Island.

Year	Total summer catch	Total autumn catch	Seasonal increase
2003	69	296	4.29
2004	33	1620	49.09
2005	1607	11712	7.29
2006	185	4347	23.50
2007	402	13673	34.01
2008	27	6843	253.44
2009	160	13046	81.54
2010	1127	3317	2.94
2011	250	1915	7.66
2012	296	682	2.30
2013	68	4446	65.38
2014	701	17791	25.38
2015	569	5132	9.02
2016	100	5226	52.26
Overall mean ±SEM, *n* = 14	399.57 ± 124.59	6431.86 ± 1461.34	44.15 ± 17.48

It is generally accepted that the emigration of insects is always initiated by sexually immature individuals, leading to the formulation of the ‘oogenesis-flight syndrome’ [71], where reproduction and long-range migration were considered alternate and ‘incompatible’ physiological states. In this study, the results of ovarian dissection showed that most of *S*. *exigua* females trapped in June (94.4 ± 10.7%) and July (80.0 ± 6.4%) (Fig 5B; S2B Fig) had mated and were sexually mature individuals (88.9 ± 11.1% in June, 61.8 ± 12.3% in July; Fig 5C; S2C Fig).

On the one hand, if these female moths emigrated far from the trapping site and therefore undertook several successive nights of long-duration flight, then our results suggested the onset of sexual maturation did not result in the termination of migration behavior of this species. The relationship between long-distance migration and the state of oogenesis appears to be similar to that of the spring population of *Agrotis ipsilon* (Rottemberg) in North America [59]. Here the poleward-moving spring migrants developed reproductively, and it was suggested that there was no need to shut down reproductive development because the movement takes place rapidly, aided by the low-level jet stream [59]. This onset of maturation during long-distance migration would be advantageous for migrating females, allowing them to initiate oviposition as soon as possible after finding a suitable habitat [72]. Similar phenomenon had been found in many lepidopteran insects, such as the spring or summer population of *Heliothis viriplaca* (Hüfnagel) [28], *Agrotis segetum* Denis and Schiffermaller [46], *Mamestra brassicae* L. [47], *Plutella xylostella* (L.) [73], and *Cnaphalocrocis medinalis* (Guenée) [74] trapped in the same site.

On the other hand, if these female moths originated not far from the trapping site, then our results indicated that the initiation of migration by *S*. *exigua* may be independent of the degree of ovarian development and mating status, providing direct field observation evidence against the generality of the ‘oogenesis-flight syndrome’. Moreover, our results also suggest that the reproductive migration of *S*. *exigua* took place not only in the summer (poleward) population but also in the autumn (returning) population.

Our observations provide a good starting point of study on the trans-regional migration of *S*. *exigua* between its overwintering and summer breeding regions. These findings contribute to a better understanding of the occurrence regularity of *S*. *exigua* migration among different climate zones, improving the forecasting of this pest. However, further studies are required to characterize the population fluctuations of this pest on the Chinese mainland. In addition, a three-dimensional trajectory analysis would be beneficial to determine the migration routes and sources of the sampled individuals.

## Supporting information

S1 Fig **Yearly (A) and monthly (B) capture of *Spodoptera exigua* moths in the searchlight trap on BeiHhuang Island from April to October 2003–2016.** Note: Vertical bars in (B) represent standard errors, and bars sharing the same letter mean there were no significant inter-month differences at the 5% level by Tukey’s HSD tests.
(TIF)

S2 Fig **Proportion of females (A), mated females (B), and sexually mature females (c) of *Spodoptera exigua* captured in the searchlight trap from June to October 2005–2009.** Note: the histograms in (A) and (B) indicate the mean proportion in each month. The histograms in (C) indicate the mean ovarian development level in each month. Vertical bars represent standard errors between days in each month.
(TIF)

S3 Fig Proportion of mating occurrences of *Spodoptera exigua* females captured in the searchlight trap on BeiHhuang Island from June to October 2005–2009. (TIF)

S4 Fig Incidence of ovarian development of *Spodoptera exigua* captured in the searchlight trap on BeiHhuang Island from June to October 2005–2009. Note: The total number of dissected *S*. *exigua* female moths was 1,724 individuals in 2005, 1,038 individuals in 2006, 1,418 individuals in 2007, 682 individuals in 2008 and 970 individuals in 2009.
(TIF)

S1 Table Two-way ANOVA analysis on the number of *Spodoptera exigua* moths captured in the searchlight trap on BeiHuang Island from May to October 2003–2016. (DOCX)

S2 Table Hierarchical clustering analysis on the annual total numbers of *Spodoptera exigua* moths captured in the searchlight trap on BeiHuang Island from May to October 2003–2016. (DOCX)

S3 Table Two-way ANOVA analysis on the monthly mean proportion of *Spodoptera exigua* females captured in the searchlight trap on BeiHuang Island from May to October 2003–2016. (DOCX)

S4 Table Two-way ANOVA analysis on the monthly mean proportion of mated *Spodoptera exigua* females captured in the searchlight trap on BeiHuang Island from May to October 2003–2016. (DOCX)

S5 Table Two-way ANOVA analysis on the monthly mean proportion of sexually mature *Spodoptera exigua* females captured in the searchlight trap on BeiHuang Island from May to October 2003–2016. (DOCX)
