# Supplementary figures and images for: Trans-regional migration of the beet armyworm, Spodoptera exigua (Lepidoptera: Noctuidae), in North-East Asia
Source: PLoS One. 2017 Aug 25;12(8):e0183582. doi: 10.1371/journal.pone.0183582 (PMC5571959; doi:10.1371/journal.pone.0183582)

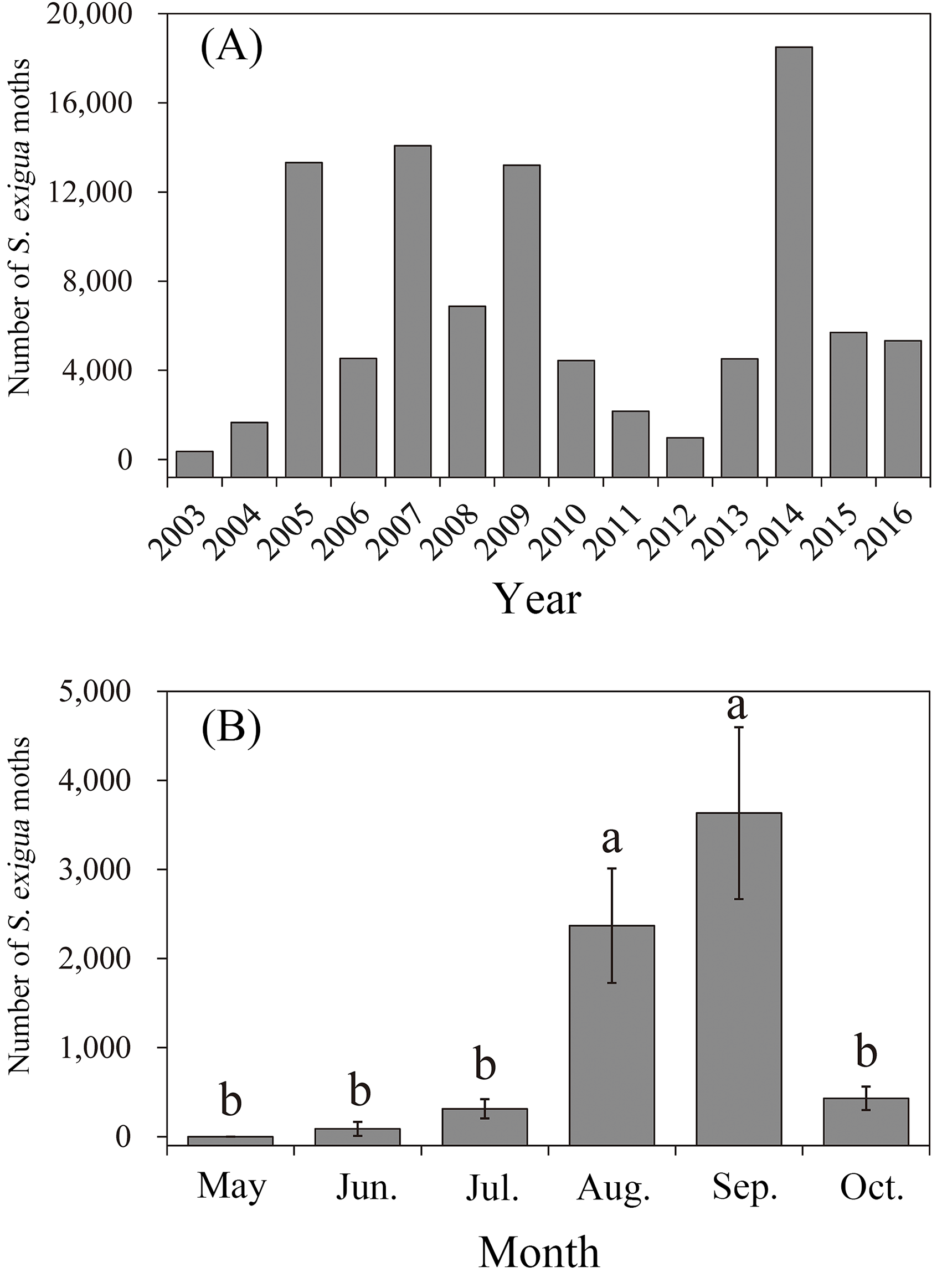

Supplement: S1 Fig — Yearly (A) and monthly (B) capture of Spodoptera exigua moths in the searchlight trap on BeiHhuang Island from April to October 2003–2016. Note: Vertical bars in (B) represent standard errors, and bars sharing the same letter mean there were no significant inter-month differences at the 5% level by Tukey’s HSD tests. (TIF) [file pone.0183582.s001.tif]

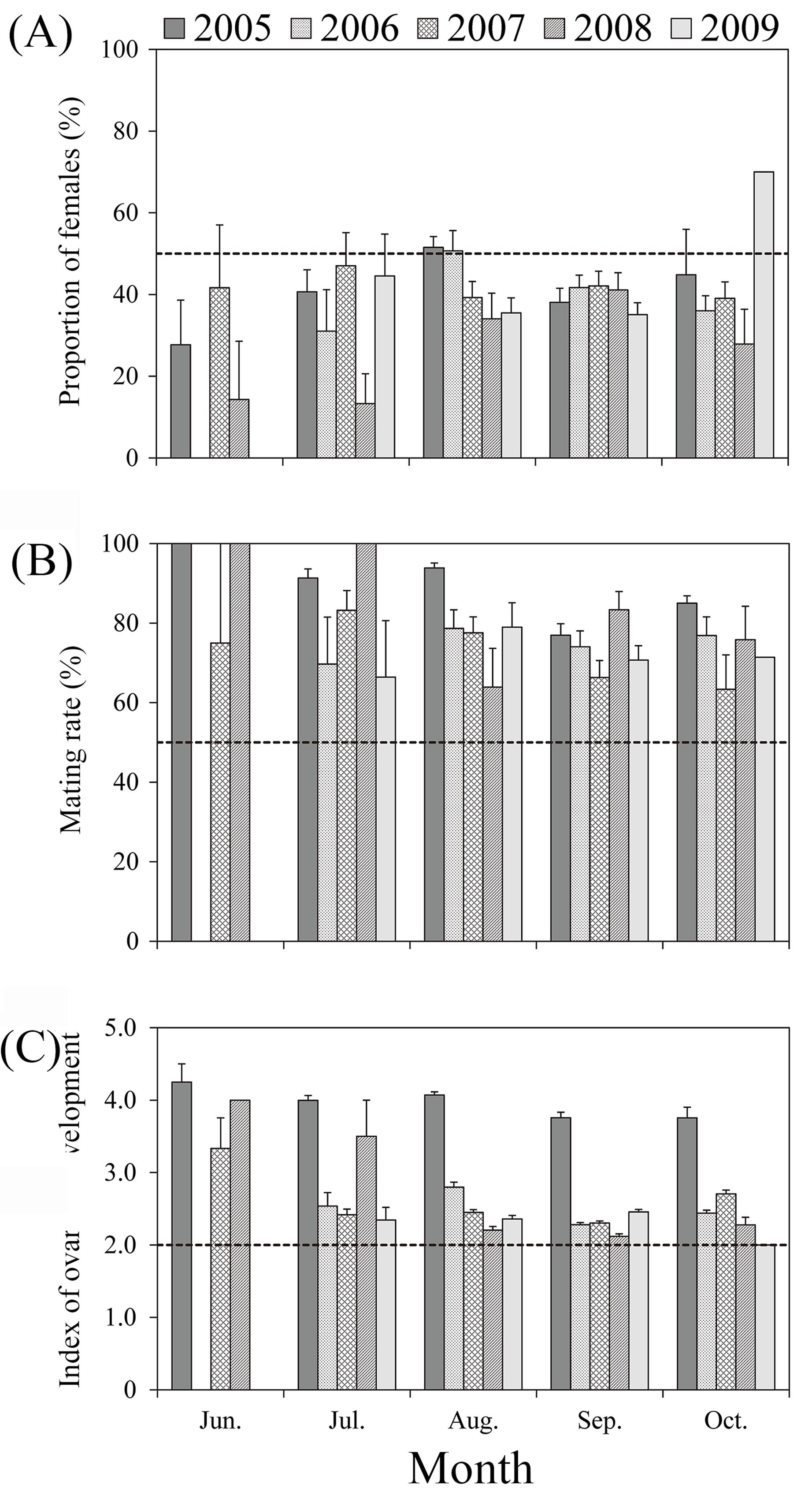

Supplement: S2 Fig — Proportion of females (A), mated females (B), and sexually mature females (c) of Spodoptera exigua captured in the searchlight trap from June to October 2005–2009. Note: the histograms in (A) and (B) indicate the mean proportion in each month. The histograms in (C) indicate the mean ovarian development level in each month. Vertical bars represent standard errors between days in each month. (TIF) [file pone.0183582.s002.tif]

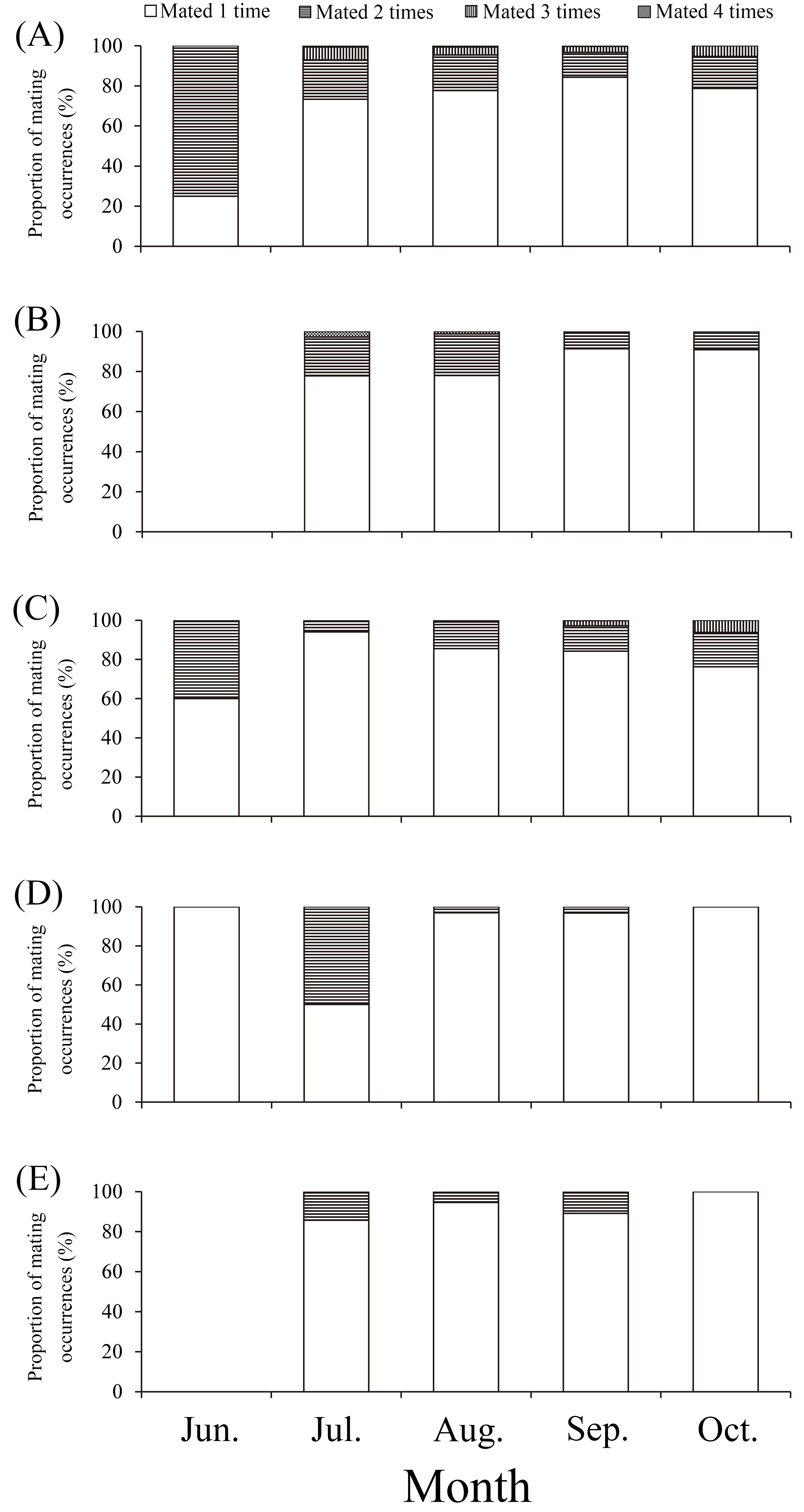

Supplement: S3 Fig — (TIF) [file pone.0183582.s003.tif]

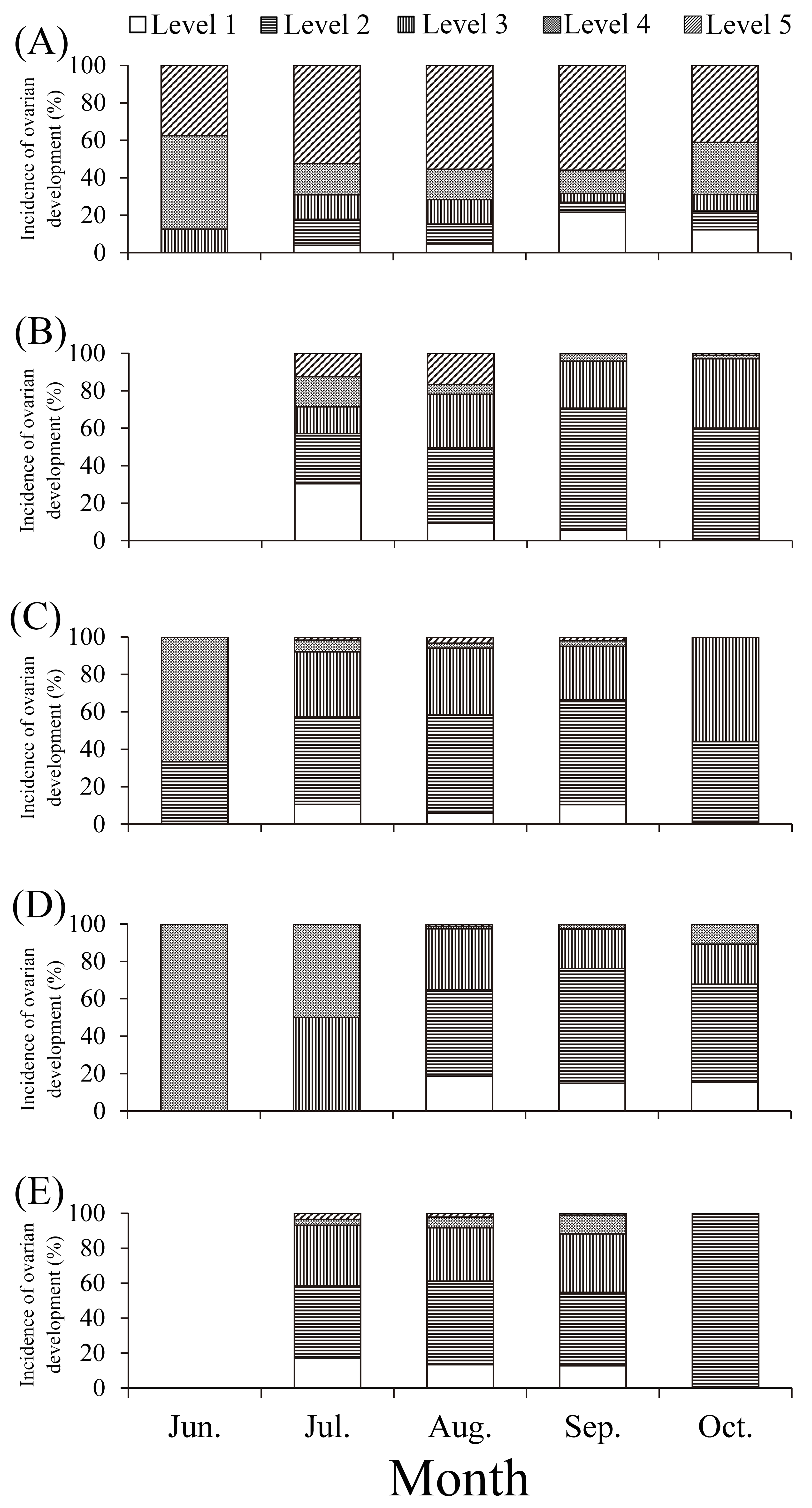

Supplement: S4 Fig — Note: The total number of dissected S. exigua female moths was 1,724 individuals in 2005, 1,038 individuals in 2006, 1,418 individuals in 2007, 682 individuals in 2008 and 970 individuals in 2009. (TIF) [file pone.0183582.s004.tif]
