# Supplementary material for: Trans-regional migration of the beet armyworm, Spodoptera exigua (Lepidoptera: Noctuidae), in North-East Asia
Source: PLoS One. 2017 Aug 25;12(8):e0183582. doi: 10.1371/journal.pone.0183582 (PMC5571959; doi:10.1371/journal.pone.0183582)
Supplement: S1 Table — (DOCX) [file pone.0183582.s005.docx]

**Table S1. Two-way ANOVA analysis on the number of *Spodoptera exigua* moths captured in the searchlight trap on BeiHuang Island from May to October 2003-2016.**

| Source | Type Ⅲ sum of squares | *df* | Mean squares | *F*-values | P |
| --- | --- | --- | --- | --- | --- |
| Month | 5560.80 | 6 | 890153.18 | 9.43 | <0.001 |
| Year | 10315.71 | 13 | 139611.91 | 3.37 | <0.001 |
| Month × Year | 50573.04 | 74 | 94409.67 | 2.28 | <0.001 |
| Error | 964810.97 | 2297 | 41456.22 |  |  |
| Total | 1036388.81 | 2390 |  |  |  |
