# Supplementary material for: Trans-regional migration of the beet armyworm, Spodoptera exigua (Lepidoptera: Noctuidae), in North-East Asia
Source: PLoS One. 2017 Aug 25;12(8):e0183582. doi: 10.1371/journal.pone.0183582 (PMC5571959; doi:10.1371/journal.pone.0183582)
Supplement: S2 Table — (DOCX) [file pone.0183582.s006.docx]

**Table S2. Hierarchical clustering analysis on the annual total numbers of *Spodoptera exigua* moths captured in the searchlight trap on BeiHuang Island from May to October 2003-2016.**

| Groups | Year | Observed value | Distance to the center | Group mean | RMSSTD |
| --- | --- | --- | --- | --- | --- |
| First group | 2014 | 18492 | 0 | 18492 | -- |
| Second group | 2005 | 13319 | 214.33 | 13533.33 | 236.24 |
|  | 2007 | 14075 | 541.67 |  |  |
|  | 2009 | 13206 | 327.33 |  |  |
| Third group | 2006 | 4532 | 699.17 | 5231.17 | 476.36 |
|  | 2008 | 6870 | 1638.83 |  |  |
|  | 2010 | 4444 | 787.17 |  |  |
|  | 2013 | 4514 | 817.17 |  |  |
|  | 2015 | 5701 | 469.83 |  |  |
|  | 2016 | 5326 | 94.83 |  |  |
| Fourth group | 2003 | 365 | 925.25 | 1290.25 | 392.68 |
|  | 2004 | 1653 | 362.75 |  |  |
|  | 2011 | 2165 | 874.75 |  |  |
|  | 2012 | 978 | 312.25 |  |  |
| *R*^2^ | 0.98 |  |  |  |  |
| Pseudo_F | 199.41 |  |  |  |  |
| *df* | (3, 10) |  |  |  |  |
| *p* | < 0.001 |  |  |  |  |
