# Supplementary material for: Trans-regional migration of the beet armyworm, Spodoptera exigua (Lepidoptera: Noctuidae), in North-East Asia
Source: PLoS One. 2017 Aug 25;12(8):e0183582. doi: 10.1371/journal.pone.0183582 (PMC5571959; doi:10.1371/journal.pone.0183582)
Supplement: S3 Table — (DOCX) [file pone.0183582.s007.docx]

**Table S3. Two-way ANOVA analysis on the monthly mean proportion of *Spodoptera exigua* females captured in the searchlight trap on BeiHuang Island from May to October 2003-2016.**

| Source | Type Ⅲ sum of squares | *df* | Mean squares | *F*-values | P |
| --- | --- | --- | --- | --- | --- |
| Month | 1582.51 | 4 | 395.63 | 0.67 | 0.626 |
| Year | 6786.74 | 4 | 1696.69 | 4.95 | < 0.001 |
| Month × Year | 8919.59 | 15 | 594.64 | 1.74 | 0.043 |
| Error | 124338.62 | 363 | 342.53 |  |  |
| Total | 143959.40 | 386 |  |  |  |
