# Supplementary material for: Trans-regional migration of the beet armyworm, Spodoptera exigua (Lepidoptera: Noctuidae), in North-East Asia
Source: PLoS One. 2017 Aug 25;12(8):e0183582. doi: 10.1371/journal.pone.0183582 (PMC5571959; doi:10.1371/journal.pone.0183582)
Supplement: S4 Table — (DOCX) [file pone.0183582.s008.docx]

**Table S4. Two-way ANOVA analysis on the monthly mean proportion of mated *Spodoptera exigua* females captured in the searchlight trap on BeiHuang Island from May to October 2003-2016.**

| Source | Type Ⅲ sum of squares | *df* | Mean squares | *F*-values | P |
| --- | --- | --- | --- | --- | --- |
| Month | 2762.96 | 4 | 690.74 | 1.27 | 0.330 |
| Year | 6203.62 | 4 | 1550.83 | 4.24 | 0.002 |
| Month × Year | 7643.71 | 14 | 545.98 | 1.49 | 0.112 |
| Error | 108236.64 | 296 | 365.66 |  |  |
| Total | 127780.73 | 318 |  |  |  |
