# Supplementary material for: Trans-regional migration of the beet armyworm, Spodoptera exigua (Lepidoptera: Noctuidae), in North-East Asia
Source: PLoS One. 2017 Aug 25;12(8):e0183582. doi: 10.1371/journal.pone.0183582 (PMC5571959; doi:10.1371/journal.pone.0183582)
Supplement: S5 Table — (DOCX) [file pone.0183582.s009.docx]

**Table S5. Two-way ANOVA analysis on the monthly mean proportion of sexually mature *Spodoptera exigua* females captured in the searchlight trap on BeiHuang Island from May to October 2003-2016.**

| Source | Type Ⅲ sum of squares | *df* | Mean squares | *F*-values | P |
| --- | --- | --- | --- | --- | --- |
| Month | 11488.39 | 4 | 2872.10 | 2.80 | 0.067 |
| Year | 28271.78 | 4 | 7067.95 | 19.09 | < 0.001 |
| Month × Year | 14376.44 | 14 | 1026.89 | 2.77 | < 0.001 |
| Error | 109574.09 | 296 | 370.18 |  |  |
| Total | 194232.70 | 318 |  |  |  |
